# Teleradiology Usage and User Satisfaction with the Telemedicine System Operated by Médecins Sans Frontières

**DOI:** 10.3389/fpubh.2014.00202

**Published:** 2014-10-28

**Authors:** Jarred Halton, Cara Kosack, Saskia Spijker, Elizabeth Joekes, Savvas Andronikou, Karen Chetcuti, William E. Brant, Laurent Bonnardot, Richard Wootton

**Affiliations:** ^1^Médecins Sans Frontières Diagnostic Network, Amsterdam, Netherlands; ^2^Epworth Medical Imaging, Epworth Freemasons Hospital, Melbourne, VIC, Australia; ^3^Department of Radiology, Royal Liverpool and Broadgreen University Hospital NHS Trust, Liverpool, UK; ^4^Department of Radiology, Faculty of Health Sciences, University of the Witwatersrand, Johannesburg, South Africa; ^5^Department of Radiology, Alder Hey Children’s Hospital, Liverpool, UK; ^6^Department of Radiology and Medical Imaging, University of Virginia Health System, Charlottesville, VA, USA; ^7^Department of Radiology, University of Colorado, Denver, CO, USA; ^8^EA 4569, Department of Medical Ethics and Legal Medicine, Paris Descartes University, Paris, France; ^9^Fondation Médecins Sans Frontières, Paris, France; ^10^Norwegian Centre for Integrated Care and Telemedicine, University Hospital of North Norway, Tromsø, Norway; ^11^Faculty of Health Sciences, University of Tromsø, Tromsø, Norway

**Keywords:** telemedicine, telehealth, teleradiology, LMICs

## Abstract

Médecins Sans Frontières (MSF) began a pilot trial of store-and-forward telemedicine in 2010, initially operating separate networks in English, French, and Spanish; these were merged into a single, multilingual platform in 2013. We reviewed the pattern of teleradiology usage on the MSF telemedicine platform in the 4-year period from April 2010. In total, 564 teleradiology cases were submitted from 22 different countries. A total of 1114 files were uploaded with the 564 cases, the majority being of type JPEG (*n* = 1081, 97%). The median file size was 938 kb (interquartile range, IQR 163–1659). A panel of 14 radiologists was available to report cases, but most (90%) were reported by only 4 radiologists. The median radiologist response time was 6.1 h (IQR 3.0–20). A user satisfaction survey was sent to 29 users in the last 6 months of the study. There was a 28% response rate. Most respondents found the radiologist’s advice helpful and all of them stated that the advice assisted in clarification of a diagnosis. Although some MSF sites made substantial use of the system for teleradiology, there is considerable potential for expansion. More promotion of telemedicine may be needed at different levels of the organization to increase engagement of staff.

## Introduction

Médecins Sans Frontières (MSF), a medical humanitarian emergency organization, operates in resource-limited settings where there are often difficulties in accessing good quality medical imaging services, such as X-ray and ultrasound. Unfortunately, there is little published information about the shortages of imaging equipment and the availability of qualified radiologists to interpret the images (1). In 2012, MSF was operating at approximately 750 locations (field sites) globally, although most did not have X-ray or ultrasound imaging facilities available on site (2). Of the sites with X-ray and ultrasound imaging available, some were MSF installed and operated, while others used local ministry of health services.

In 2010, MSF began a pilot project to provide its field sites (places where MSF provides health care services) with access to a network of specialists through a store-and-forward telemedicine platform based on the Collegium Telemedicus system (3). The system initially operated separate networks for English, French, and Spanish users, but these were merged into a single, multilingual platform in 2013. The MSF telemedicine (tele-expertise) system provides field doctors with access to a very wide range of specialists, including radiologists.

### How the system works

The telemedicine system requires field site users to log onto a secure website to submit a case that includes a brief patient history and anonymized images from different modalities. Typically, these images are from general X-ray or ultrasound examinations.

Two different types of images can be sent for teleradiology: files in DICOM format (Digital Imaging and Communications in Medicine) or files in JPEG format. Sites with access to digitally acquired images are able to upload DICOM or JPEG files exported from the medical imaging device. Sites using traditional methods of X-ray development, i.e., film and chemistry developing, are provided with a protocol for creating a digital file by photographing X-rays of acceptable quality (4, 5). The protocol for digitizing X-rays requires a digital camera of at least 3.5 Mpixel resolution, with optical image stabilization (settings: compression high, flash off, autofocus, exposure compensation set manually to +1.3 EV for chest X-rays, 0 for other anatomical areas) fixed to a tripod placed 70 cm from the light box perpendicular to the film with extraneous light blocked from the outer edges of the X-ray (4, 5).

Once a case has been submitted, an email prompt is sent automatically to the coordinators of the system who can then log on to the website and allocate the case. The allocation of a case to a radiologist for consultation is referred to as a query. Some cases require allocation to more than one radiologist, e.g., if that person is unavailable for some reason. Therefore, there are more queries than cases. An email prompt is sent to the radiologist once a case is allocated to them, and a subsequent email prompt is sent to the field site when the radiologist has submitted their findings. All specialists’ findings are purely advisory, and the final decision on patient management remains with the clinician in the field. In all cases, images, findings, and correspondence are stored securely on the telemedicine platform for ease of future reference. Patient data and images are stored on the secure website but are not included in email messages for reasons of confidentiality.

### Objective

The aim of the present study was to assess teleradiology usage on the MSF telemedicine platform since its inception. Our hypothesis was that there would be an overall growth in teleradiology usage.

## Materials and Methods

We conducted a retrospective analysis of all cases sent for radiologist consultation from April 2010 to March 2014 from data extracted from the MSF telemedicine system. Ethics permission was not required, because patient consent to access the data had been obtained and the work was a retrospective chart review conducted by the organization’s staff in accordance with its research policies.

We collated the extracted data on a spreadsheet (Excel 2007, Microsoft) and analyzed the number and origin of cases, turnaround times, the number of cases read by participating radiologists, the number of queries per case, and overall usage. Any cases apparently representing statistical outliers were reviewed individually to ascertain the reason.

Starting in October 2013, requests were sent to field staff referring cases to complete a user feedback questionnaire. The requests were sent 21 days after the referral was made. The questionnaire contained 12 questions, part of a larger study to be reported elsewhere. The present work considers the two questions relating to user satisfaction:

(1) Did you find the advice helpful?
(2) If YES, did it- (tick any that apply)
  Clarify your diagnosis
  Assist with your management of the patient
  Improve the patient’s symptoms
  Improve function
  Any other reason? Please specify

## Results

### Number of cases

In total, 564 teleradiology cases were sent in the 4-year period from April 2010 to March 2014. The first case was submitted in June 2011, so the mean referral rate from that time was approximately 16 cases per month. The maximum number of cases sent was 64, in October 2012. There was no clear pattern of overall growth in usage during the study period.

### Images

A total of 1114 files were uploaded with the 564 cases submitted. The majority of the uploaded files were of type JPEG (*n* = 1081, 97%); there were 18 compressed files (.zip), which were also mainly JPEG type (Table 1). The average number of uploaded files per case was 1.98 (range 1–16). The median file size was 938 kb (IQR 163–1659).

**Table 1:** **Contents of the compressed files**.

Case number	Content of zip file	Type of image
20	1 JPEG	Document image
419	5 JPEGs	CT scan
537	25 JPEGs	CT scan
538	16 JPEGs	CT scan
577	6 JPEGs	CT coronary scan
	2 JPEGs	Document images
	1 JPEG	Document image
	2 JPEGs	Document images
	1 JPEG	Document image
	5 JPEGs	Document image
659	53 JPEGs	MRI scan
814	7 JPEGs	Ultrasound scans
903	3 JPEGs	X-rays
904	4 JPEGs	X-rays
919	75 JPEGs	CT scan
	73 JPEGs	CT scan
1242	DICOM dataset	MRI scan
	DICOM dataset	MRI scan

### Number of queries

**Figure 1:**
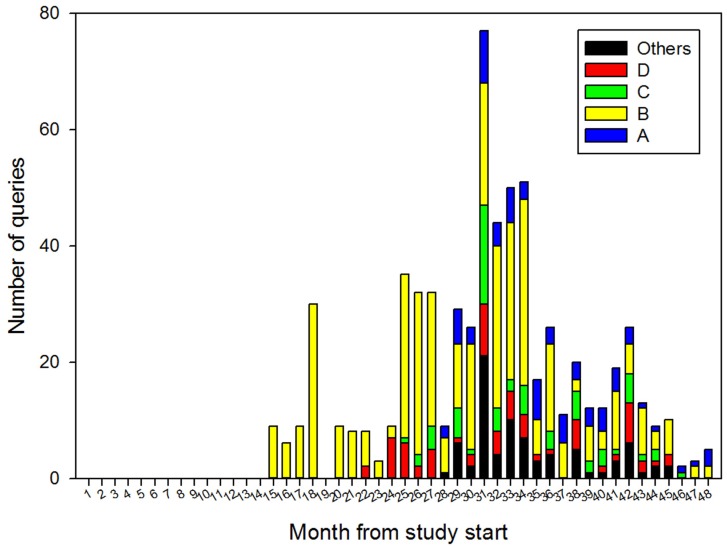
**The number of queries sent to individual radiologists (A–D) each month**.

In total, there were 661 queries from the system for a radiologist consultation. This represented an average of 1.2 queries per case. The number of queries varied between radiologists from 0 to a maximum of 32 per month for the busiest, see Figure 1.

### Origin of cases

A total of 564 cases were sent from 22 different countries (see Table 2). The majority of cases, 69% (388/564), came from two countries: Central African Republic and Malawi.

**Table 2:** **The country of origin of cases sent for teleradiology**.

Country	No. of cases
Afghanistan	1
Kyrgyzstan	1
South Africa	1
Swaziland	1
Syria	1
Georgia	2
Turkey	2
Kenya	2
Yemen	2
France (operational center)*	3
Democratic Republic of the Congo	4
Netherlands (operational center)*	4
Sudan/South Sudan	4
Armenia	5
Ethiopia	5
Guinea	12
Chad	18
Cambodia	23
Uganda	35
Tajikistan	50
Malawi	170
Central African Republic	218
Total	564

*The small number of cases from France and the Netherlands were submitted by headquarters staff on behalf of field doctors in unidentified countries.

### Turnaround time

The median delay in allocating a case by the system coordinators was 0.4 h (IQR 0.1–1.3). The median radiologist response time, which includes the delay in allocation, was 6.1 h (IQR 3.0–20), which is based on 563 cases. One additional case was allocated, but left unanswered and was excluded from the turnaround time results.

### Radiologists

**Figure 2:**
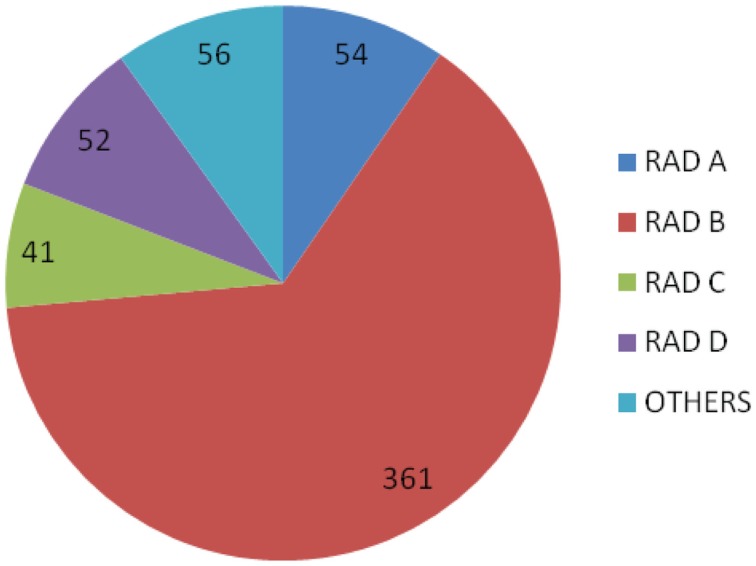
**The number of cases sent to individual radiologists (A–D)**.

A total of 14 radiologists were available through the system and during the study period 12 radiologists were sent at least one case. Four radiologists were allocated 90% (507/564) of all cases, with radiologist B allocated the majority, 64% (361/564), Figure 2.

### User satisfaction

Between 27 October 2013 and 31 March 2014, a total of 29 requests for completion of the survey were sent to referrers who had submitted radiology cases. A total of eight completed surveys were received (28% response rate). Most respondents (7/8) found the radiologist’s advice helpful and all (6/6) stated that the advice assisted in clarification of a diagnosis, see Table 3.

**Table 3:** **User satisfaction survey results**.

Case_no	Q1	Q2a	2b	2c	2d	2e
	
	Did you find the advice helpful?	Clarify your diagnosis?	Assist with your management of the patient?	Improve the patient’s symptoms?	Improve function?	
869	Yes	Yes				
890	Yes	Yes	Yes	Yes	Yes	
892	Yes					In this case, we were asking a general question regarding the quality of the X-ray (lateral in a child). We received very clear advice, as well as additional advice on the use of lateral X-ray in kids
919	No					
929	Yes	Yes	Yes	No	No	
933	Yes	Yes	Yes			
934	Yes	Yes	Yes			
1226	Yes	Yes	Yes	No	No	All that was requested was an interpretation of a CT scan (thus advice did not impact function or symptoms)
Total	7/8 Yes	6/6 Yes	5/5 Yes	1/3 Yes	1/3 Yes	

## Discussion

### Number of cases

Teleradiology via the MSF Telemedicine system has been possible since April 2010, although it took over a year for the first teleradiology case to be sent in June 2011. The lack of system usage during that time was probably due to a lack of awareness within field sites and operational centers, the time taken to acquire acceptance of a new technology, and the limited number of field sites with access to both medical imaging facilities and a suitable Internet connection. The increase in cases requesting radiology advice occurred after a focused effort by the MSF Diagnostic Imaging Working Group to promote the service among headquarters and field staff through emails, internal newsletters, presentations at internal scientific events and workshops.

### Origin of cases

Médecins Sans Frontières has been operating in Malawi and Central African Republic for over 20 years (6, 7). Recent collaborations on the use of teleradiology in Malawi (8) and an X-ray installation with mandatory teleradiology use in Central African Republic probably explain the high number of cases from these countries.

### Usage

**Figure 3:**
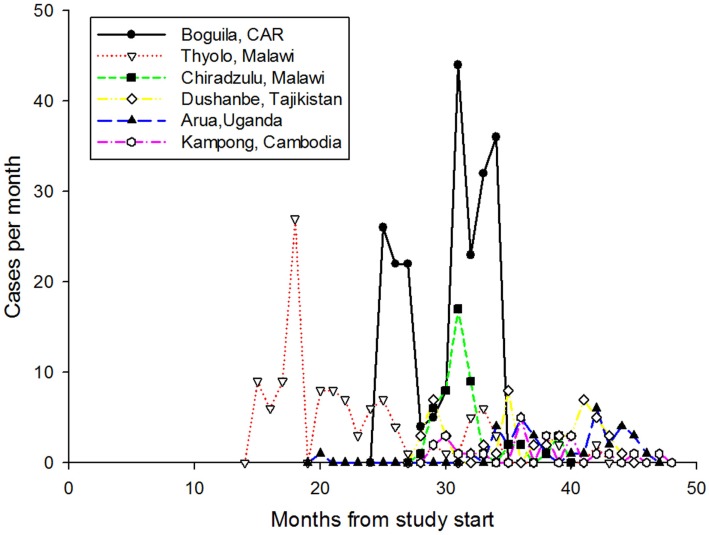
**The pattern of usage from the six sites sending the most cases**. During the study, these sites submitted 496 cases.

Although some field sites made substantial use of the system for teleradiology, there is clearly considerable potential for expansion. There were also fluctuations in the use of the system, which can probably be attributed to changes in personnel at field sites familiar with the telemedicine system, limitations to Internet access in remote locations, and periods of medical equipment repair/maintenance. The pattern of usage for the six sites referring the most cases showed no general trends (see Figure 3). A number of sites sent fewer than 10 cases per month with irregular, unexplained peaks, and troughs. The period from April 2012 to January 2013 showed relatively consistent usage with the peak volume of teleradiology cases occurring in October 2012. This coincided with the field site in Central African Republic commencing use of the system, which continued until the end of January 2013. The usage stopped in January 2013 due to the deterioration of the security situation and removal of most expatriate staff from Boguila in the Central African Republic. This also explains in part the decreases in total number of cases for the period February 2013 to March 2014.

### Images

A quality assessment of all X-rays sent for teleradiology in 2012 for MSF reported a clear superiority in the quality of X-rays originating from sites with computed radiography (CR) compared with traditional film chemistry development (9). This assessment included the MSF Telemedicine system and a separate platform, vRad (Virtual Radiologic Corporation, Eden Prairie, MN, USA), a commercial teleradiology company providing *pro bono* services to MSF.

### Number of queries

The difference between the number of cases (554) and the number of queries (661) was caused by certain cases requiring a second or multiple allocations to different radiologists. This may occur in situations when the radiologist first allocated a case was not available to reply within 24 h, the allocation was made to an incorrect sub specialist (for example, adult images being sent to a pediatric radiologist), or the expertise of more than one radiologist was required for a single case.

During the evolution of the system, detailed information about radiologist availability and sub specialty expertise was acquired to guide coordinators in suitably allocating cases. This has improved the turnaround times.

### Turnaround time

The telemedicine system is available 24 h a day, 7 days a week and cases are submitted, allocated, and reviewed from different countries and time zones around the world. Cases are allocated based on the clinical question raised by the field worker, type of imaging study, sub specialty of the radiologist, and the time zone the radiologist reporting the cases operates from. The system is not designed for life-threatening emergency cases, since it operates in store-and-forward mode.

### Radiologists

The system has relied heavily on four radiologists who were consulted in the majority of cases (90%). This has been beneficial for continuity and familiarity between the field site staff and the radiologist, and the radiologist’s understanding of available resources and typical disease burdens at that site. However, this is also a potential disadvantage in the future, especially if the volume of cases increases, as it may create an over-reliance on a small number of radiologists and loss of interest from other less active radiologists. Recruitment of additional radiologists is hampered by the requirement to understand local disease epidemiology and health care setting. Radiologists working in low resource settings are scarce and less likely to be available for teleradiology support, in addition to their daily workload.

### Lessons learnt

Despite the system being available to all MSF field sites with access to medical imaging facilities and despite very few radiologists being present in the field, only a limited number of sites have made regular use the service. There has been no overall trend of increased use of the system. There could be a number of reasons for this, including a lack of knowledge about the availability of teleradiology. A regular program of information about the system might be helpful in the future. Periods of peak usage were directly related to particular sites or individual referrers, rather than a collective increase across numerous sites. Briefing of expatriate staff prior to departure for a field site, and promotion of use by medical advisers at headquarters, would be beneficial in ensuring the continued expansion of the service.

### Limitations

The system is easy to use for all those involved. However, due to the high turnover of field staff, certain problems have occurred, such as login details (username and password) being lost or forgotten and being re-requested frequently, and images not being uploaded with a case. In addition, there was one unanswered query that was overlooked at the end of a long thread of patient discussion. User satisfaction surveys are a recent addition and only a small number of cases were included.

## Conclusion

The multilingual telemedicine system has been used successfully by several field sites for teleradiology, with a total of 564 cases submitted, at a mean rate of about 16 cases per month. The median response time was 6.1 h. Most field users found the radiologist’s advice helpful and stated that the advice assisted in clarification of a diagnosis. Despite the system being available to all MSF field sites with access to medical imaging facilities, there has been no overall growth in use of the system for teleradiology. More promotion of telemedicine may be needed at different levels of the organization to increase engagement of staff.

## Conflict of Interest Statement

The authors declare that the research was conducted in the absence of any commercial or financial relationships that could be construed as a potential conflict of interest.

## References

[1] Afro.who.int. Overview – WHO | Regional Office for Africa. [online]. (2014). Available from: http://www.afro.who.int/en/clusters-a-programmes/hss/blood-safety-laboratories-a-health-technology/overview.html

[2] Etchegorry M Neerkorn J Médecins Sans Frontières (MSF) International. 2012 Operational analysis – MSF International Typology. Internal Document (2014).

[3] Wootton R Wu W Bonnardot L. Nucleating the development of telemedicine to support healthcare workers in resource-limited settings: a new approach. J Telemed Telecare (2013) 19(7):411–7. 10.1177/1357633X13506511 24218356

[4] Corr P Couper I Beningfield SJ Mars M. A simple telemedicine system using a digital camera. J Telemed Telecare (2000) 6(4):233–6. 10.1258/1357633001935293 11027126

[5] Szot A Jacobson FL Munn S Jazayeri D Nardell E Harrison D Diagnostic accuracy of chest X-rays acquired using a digital camera for low-cost teleradiology. Int J Med Inform (2004) 73:65–73. 10.1016/j.ijmedinf.2003.10.002 15036080

[6] Médecins Sans Frontières (MSF) International. International Activity Report 2012 – Malawi. [online]. (2014). Available from: http://www.msf.org/international-activity-report-2012-malawi

[7] Médecins Sans Frontières (MSF) International. International Activity Report 2012 – Central African Republic. [online]. (2014). Available from: http://www.msf.org/international-activity-report-2012-central-african-republic

[8] Coulborn R Panunzi I Spijker S Brant W Duran L Kosack C Feasibility of using teleradiology to improve tuberculosis screening and case management in a district hospital in Malawi. Bull World Health Organ (2012) 90(9):705–11. 10.2471/BLT.11.099473 22984316 PMC3442390

[9] Spijker S Andronikou S Kosack C Wootton R Bonnet M Lemmens N. Quality assessment of X-rays interpreted via teleradiology for Médecins Sans Frontières. J Telemed Telecare (2014) 20(2):82–8. 10.1177/1357633X14524153 24518926

